# First case of primary CNS lymphoma in a patient with severe combined immunodeficiency carrying a novel ZAP70 mutation: a case report

**DOI:** 10.1097/MS9.0000000000003591

**Published:** 2025-07-15

**Authors:** Mohammed Hady Albitar, Ahmed Gamal Sayed, Faisal Joueidi, Nida Mariyam, Maeen Bassam AlDamouni, Waad Ahmed Albalawi, Rand Arnaout

**Affiliations:** a College of Medicine, Alfaisal University, Riyadh, Saudi Arabia; b Department of Neurology/Internal Medicine, Security Forces Hospital, Makkah, Saudi Arabia; c Department of Allergy and Immunology, King Abdulaziz Medical City, Riyadh, Saudi Arabia; d Department of Medicine, Section of Allergy/Immunology, King Faisal Specialist Hospital & Research Center, Riyadh, Saudi Arabia

**Keywords:** ZAP70, SCID, diffuse large B-cell lymphoma, immunodeficiency, Epstein–Barr virus (EBV)

## Abstract

**Introduction:**

Zeta-chain-associated protein kinase 70 (*ZAP70*) is a tyrosine kinase that plays a crucial role in T-cell activation via the T-cell receptor/CD3 complex and contributes to B-cell signaling. *ZAP70* variants can cause a range of immunodeficiencies with variable clinical presentations, including infections and malignancies.

**Case Presentation:**

A 4-year-old boy presented with chronic cough, dyspnea, recurrent chest infections, and failure to thrive. Chest radiography revealed diffuse bilateral opacities, suggesting a diagnosis of diffuse familial bronchiectasis. Immunological workup at 18 years of age showed CD4^+^ and CD8^+^ T-cell lymphopenia, and genetic analysis revealed a homozygous pathogenic *ZAP70* splice-site mutation (c.402 + 2 T>C). The patient then developed headaches, dizziness, and double vision, and Epstein–Barr virus (EBV) polymerase chain reaction (PCR) testing revealed a viral load of 700 IU/mL. Magnetic resonance imaging (MRI) revealed three brain lesions, and brain biopsy confirmed EBV-positive CD20 + diffuse large B-cell lymphoma (DLBCL). Despite aggressive chemotherapy and palliative radiotherapy, the patient’s condition deteriorated, resulting in death.

**Discussion:**

To our knowledge, this is the first known case of primary central nervous system DLBCL in a patient with a novel *ZAP70* variant. *ZAP70* deficiency is typically associated with combined immunodeficiency and rarely with malignancies such as leukemia or lymphoma. Genetic screening at earlier stages could have potentially identified this underlying immunodeficiency sooner and altered the management course.

**Conclusion:**

This case underscores the diagnostic challenges and aggressive course of *ZAP70*-related disease and highlights the need for increased clinical suspicion, immunologic surveillance, early genetic screening, and development of targeted therapies.

## Introduction

*ZAP70* is a 70-kDa tyrosine kinase encoded by the *ZAP70* gene on chromosome 2q11.2 and is primarily expressed on αβ and γδ T cells, natural killer (NK) cells, and certain B-cell subsets^[1,2]^. *ZAP70* plays a key role in T-cell activation through its interaction with the ζ-chain of the TCR/CD3 complex^[3,4]^. As a member of the Syk kinase family, *ZAP70* is also involved in multiple complex processes in B-cell activation and signaling via the B-cell transduction complex^[5,6]^.

**HIGHLIGHTS**

- *ZAP70* deficiency is a rare autosomal recessive form of SCID that causes defective T-cell signaling and recurrent infections.
- This is the first reported case of EBV-associated primary CNS DLBCL in a patient with a novel homozygous *ZAP70* splice-site mutation (c.402 + 2 T>C).
- Initial presentation with bronchiectasis and delayed diagnosis until age 18 years despite long-standing immunodeficiency.
- The patient received MATRIX chemotherapy, corticosteroids, antivirals, IVIG, and palliative whole-brain radiotherapy.
- This case highlights the broad clinical spectrum of *ZAP70* deficiency and underscores the importance of early genetic testing for atypical cases.

Various mutations in *ZAP70* have been reported, including complete loss of expression, hypomorphic mutations, and compound heterozygous variants^[7]^. These mutations can lead to loss or gain of function, resulting in primary immunodeficiency, such as common variable immunodeficiency (CVID) and severe combined immunodeficiency (SCID)^[8]^. SCID is associated with mutations in more than 30 genes, and genetic testing is required to identify the underlying mutation^[9]^. Additionally, *ZAP70* mutations have also been implicated in B-cell and lymphoid malignancies, including chronic lymphocytic leukemia (CLL) and some types of lympomas^[10,11]^. We describe the first reported case of EBV-associated primary CNS lymphoma (PCNSL) in a patient with a novel *ZAP70* mutation. This case reinforces the need for increased clinical suspicion and standardized immunologic screening in children with recurrent infections, especially when accompanied by a suggestive family history or poor response to standard treatments. This case was reported in line with the SCARE 2025 criteria^[12]^.

## Case presentation

The patient initially presented to our tertiary care hospital at the age of 4 years with chronic productive cough associated with greenish sputum, exertional dyspnea, recurrent chest infections, tinea corporis, and failure to thrive. Examination revealed nail clubbing, weight and height below the 10th percentile, and diminished breath sounds on the right side with bilateral crepitations. Examination of other systems was unremarkable. Chest radiography showed diffuse bilateral opacities, and *Haemophilus influenzae* was isolated from sputum culture. The patient was diagnosed with diffuse familial bronchiectasis and an abnormal T-cell blastogenesis. Both of these findings were suggestive of T-cell dysfunction. The family history was significant for a brother who died of a lymphoproliferative disorder. The patient was then managed with prophylactic azithromycin (250 mg daily for 3 days), fluticasone/salmeterol (two puffs twice daily), and regular chest physiotherapy.

Initial immune evaluation using flow cytometry revealed CD3^+^ T cells at 35%, CD4^+^ T-helper cells at 741/µL, CD8^+^ T-cytotoxic cells at 682/µL, and CD19^+^ B cells at 638/µL. At that time, these results were near the lower limit of the normal range and did not prompt further investigation.

By the age of 18 years, routine laboratory testing revealed a marked decline in T-cell subsets: CD3^+^ at 27%, CD4^+^ at 59/µL, CD8^+^ at 19/µL, and absolute lymphopenia. This prompted a comprehensive genetic evaluation. Targeted next-generation sequencing (NGS) was performed using an Illumina-based platform, covering the coding regions and flanking introns of known immune-related genes. The variants were aligned to the human genome reference GRCh37/hg19 and filtered using public and proprietary databases. A homozygous splice-site mutation in *ZAP70* (NM_001079: exon 3, c.402 + 2 T>C) was indentified, disrupting a conserved donor site. This variant was classified as likely pathogenic (ACMG Class 4) and was confirmed by Sanger sequencing^[11]^.

**Figure 1.**
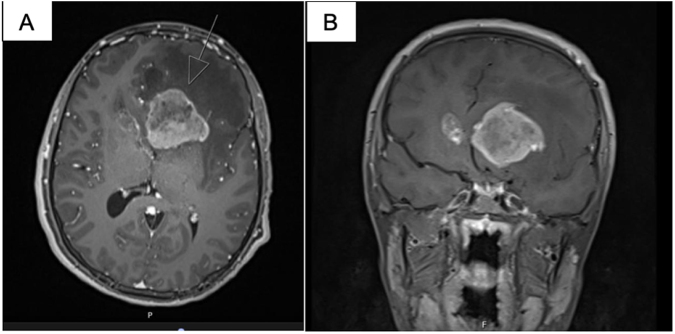
T1-weighted MRI showing a periventricular mass (41 × 32 × 13 mm) near the left frontal horn with an associated contralateral nodule in the caudate head (14 × 13 x 17 mm), and a 6 mm nodule in the right cerebellar hemisphere (a, axial; b, coronal).

**Figure 2.**
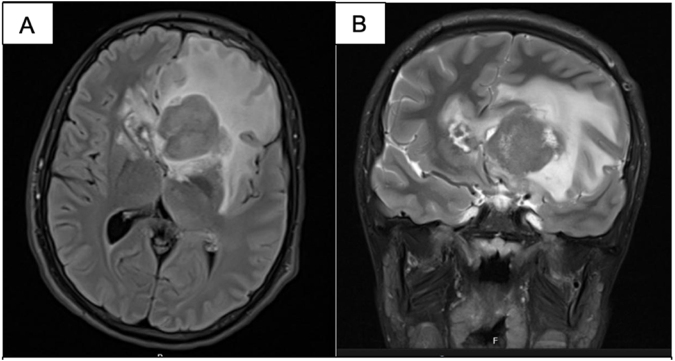
T2/fluid-attenuated inversion recovery (FLAIR) imaging showing subcortical non-enhancing hyperintensity in the frontal lobe extending into the genu of the corpus callosum and into the intervening falx (a, axial; b, coronal).

Two weeks later, the patient developed generalized headache, diplopia, dizziness, left lid lag, and sixth cranial nerve palsy. Epstein–Barr virus (EBV) polymerase chain reaction (PCR) revealed a viral load of 700 IU/mL. Brain magnetic resonance imaging (MRI) revealed a 41 × 32 × 13 mm periventricular mass near the left frontal horn of the lateral ventricle, a 14 × 13 × 17 mm contralateral nodule in the caudate head, and a 6 mm nodule in the peri-dentate area of the right cerebellar hemisphere (Fig. 1a, b). No invasion of the corpus callosum or falx cerebri was noted. T2-weighted gadolinium-enhanced MRI revealed peripheral cystic degeneration. T2/FLAIR imaging showed subcortical non-enhancing hyperintensity in the frontal lobe extending into the genu of the corpus callosum (Fig. 2a, b). The paranasal sinuses, orbits, mastoid air cells, and the brainstem were unremarkable.

**Figure 3.**
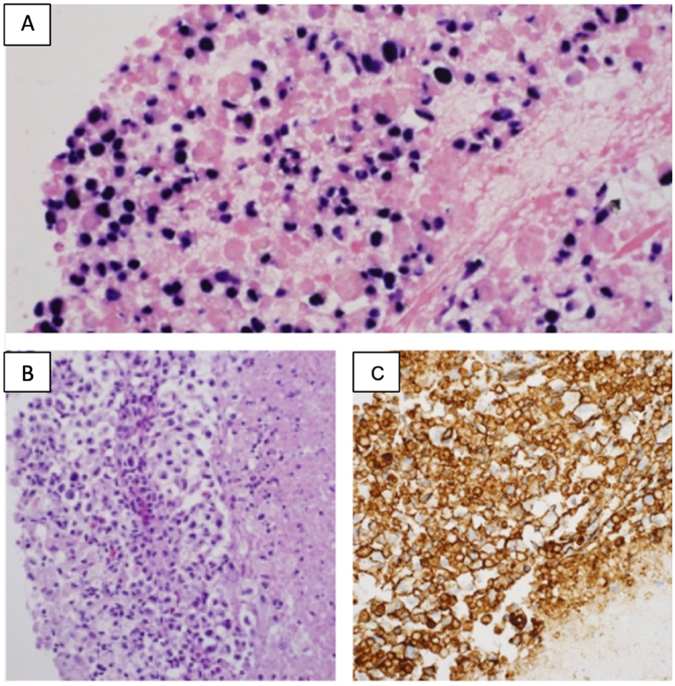
(A) Sheets of large tumor cells with necrosis and scattered viable cells (H&E, 400×). (B) Strong and diffuse membranous CD20 positivity in tumor cells (IHC, 400×). (C) *In situ* hybridization demonstrating EBV DNA within the tumor cells (400×).

Histopathology of the brain lesion revealed extensive necrosis interspersed with large viable neoplastic cells characterized by vacuolated cytoplasm and prominent nucleoli (Fig. 3a). Immunohistochemistry revealed strong diffuse membranous CD20 positivity (Fig. 3b), MUM1 and BCL2 expression, and negative staining for BCL6, CD10, GFAP, OLIG2, and IDH-1. The Ki-67 proliferation index was approximately 30%. *In situ* hybridization (EBER-ISH) revealed EBV-positive tumor cells (Fig. 3c), confirming the diagnosis of EBV-associated primary CNS diffuse large B-cell lymphoma (DLBCL).

The patient received three cycles of MATRIX (methotrexate, cytarabine, thiotepa, and rituximab) chemotherapy, along with corticosteroids, antiviral agents, and intravenous immunoglobulin (IVIG). Following the first chemotherapy cycle, the patient developed febrile neutropenia secondary to gram-negative bacteremia. The patient was initially treated with cefepime and later switched to meropenem. PCR showed cytomegalovirus (CMV) viremia of 6,299 IU/mL. The second cycle was complicated by prolonged cytopenia, requiring supportive transfusions and granulocyte colony-stimulating factor (G-CSF). Follow-up MRI showed reduced lesion sizes and stable hemorrhagic areas.

During the third cycle, the patient developed jerky movements on the left side, which progressed to a generalized tonic-clonic seizure lasting 2–3 min, wich was aborted with lorazepam. Subsequently, the patient was maintained on levetiracetam. Electroencephalography (EEG) revealed periodic lateralized epileptiform discharges in the right temporal region. Computed tomography (CT) showed increased right temporal hemorrhage, worsening edema, and midline shift. The left frontal lesion decreased in size to approximately 24 × 14 mm, whereas the right lesion enlarged to approximately 25 × 24 mm. One week later, the patient underwent a right temporal craniotomy with hematoma evacuation, lesion biopsy, and external ventricular drain (EVD) placement. Postoperative CT showed slight improvement in the mass effect and midline shift. Histopathological testing confirmed a persistent active EBV-positive DLBCL.

Given the refractory disease course and evident disease progression after three chemotherapy cycles, palliative whole-brain radiotherapy (20 Gy in five fractions) was initiated, following multidisciplinary discussion and in full agreement with the family as curative options were no longer viable. Radiotherapy was offered solely for symptom control, particularly to relieve the elevated intracranial pressure. The patient’s hospital course was later complicated by aspiration pneumonia, increased intracranial pressure, and seizures. The patient passed away 2 days after the initiation of radiotherapy.

## Discussion

*ZAP70* mutations cause T-cell immunodeficiency by disrupting TCR signaling^[11]^. Infants with *ZAP70* deficiency typically present with recurrent infections, such as severe respiratory tract infections and oral candidiasis, along with failure to thrive^[8,11]^. Classically, *ZAP70*-related combined immunodeficiency is characterized by normal CD3^+^ counts, normal or elevated CD4^+^T cells, and markedly reduced or absent CD8^+^ T-cells (0–2% of total T cells). Management involves administration of intravenous immunoglobulin and prophylaxis against viral, bacterial, and fungal infections^[11,13]^. Without hematopoietic stem cell transplantation, survival beyond 2 years is uncommon^[4]^. However, some patients may have detectable lymphoid tissues, normal lymphocyte count, or overlapping features with more common respiratory disorders, such as bronchiectasis or cystic fibrosis, which can delay diagnosis^[13]^. In our case, diagnosis was delayed until the age of 18 years despite longstanding respiratory symptoms as laboratory results at that time were near the lower limit of the normal range and did not prompt further investigation.

The patient was found to carry a homozygous *ZAP70* splice-site variant (c.402 + 2 T>C), which was previously reported in a patient with primary ciliary dyskinesia^[14]^. This variant has been inconsistently reported in literature. Although initially reported as Class 2 (uncertain significance) by CENTOGENE, this designation likely reflects the limited phenotypic correlation or absence of functional validation at the time of reporting. However, based on current ACMG/AMP guidelines, the variant fulfills the criteria for reclassification as Class 4 (likely pathogenic)^[11]^. Specifically, it meets the criteria for PVS1 (Pathogenic Very Strong 1), as it disrupts a canonical splice donor site and is predicted to cause loss of function in *ZAP70*, a mechanism well-established in *ZAP70*-related SCID^[4]^. The variant was detected in homozygous form and occurred in the context of profound T-cell lymphopenia and clinical features consistent with SCID, further supporting its pathogenicity^[8]^.

Other pathogenic *ZAP70* variants have been linked to autosomal recessive conditions, such as infantile-onset multisystem autoimmune disease type 2 and immunodeficiency type 48^[15]^. Although the patient survived beyond infancy, he fulfilled the SCID diagnostic criteria according to the Primary Immune Deficiency Treatment Consortium (PIDTC) criteria definition, based on the presence of a confirmed pathogenic *ZAP70* variant, severely reduced CD4^+^ and CD8^+^ counts (<300 cells/µL), and a clinical history of recurrent infections and growth failure^[8]^. The genetic sequencing output exceeded a quality score of 350, and the finding was confirmed by Sanger sequencing. The delayed diagnosis and partially retained immune function suggest an atypical SCID phenotype, as described in other hypomorphic or late-onset *ZAP70* mutations^[11,13]^.

*ZAP70* is known to influence the tumor microenvironment by mediating interactions between malignant B cells and the surrounding immune cells^[16]^. In this case, the patient developed primary central nervous system lymphoma (PCNSL), which was histologically consistent with DLBCL, highlighting an increased susceptibility to viral complications, particularly EBV viremia, which is commonly associated with PCNSL^[7,17]^.

EBV is a globally prevalent virus that primarily infects B cells and commonly causes infectious mononucleosis^[18]^. In immunocompromised hosts, EBV can impair T-cell responses, leading to blast cell proliferation and the development of lymphoma or lymphoproliferative disease^[17]^. *ZAP70* plays a key role in natural killer (NK) cell development and is required to activate T cells and dendritic cells via IL-2 and IFN-γ responses, all of which are crucial for EBV control^[19]^. Additionally, impaired T-cell recognition of EBV antigens may reduce host resistance to EBV-infected cells^[17,18]^. In this patient, the EBV load was modest (700 IU/mL); however, CNS lymphoma was already advanced, suggesting that low-level viremia can trigger lymphomagenesis in patients with severe T-cell dysfunction^[17,18]^. Similar cases have been documented in hypomorphic *ZAP70* mutations, in which relatively low or fluctuating EBV viral loads are sufficient to precipitate lymphoproliferative disorders due to underlying qualitative immune defects^[8,11]^. The broader literature on EBV-driven disease in primary immunodeficiency supports the concept that impaired cellular immunity, rather than viral burden alone, determines malignancy risk^[17–20]^.

Different types of malignancies have been reported in patients with *ZAP70* deficiency. In a systematic review of 49 patients, Sharifinejad et al reported malignancy in 8.1% of cases, predominantly lymphomas and leukemias^[8]^. Shirkani et al. reported a case of *ZAP70* that presented with severe infections and later developed multisystem autoimmunity and lymphoproliferation without a confirmed malignancy^[11]^. Another report by Kaman et al. described compound heterozygous *ZAP70* mutations resulting in a SCID phenotype without an associated malignancy^[1,2]^. To our knowledge, this is the first reported case of PCNSL in a patient with *ZAP70* deficiency.

PCNSL is a rare and aggressive extranodal non-Hodgkin lymphoma that accounts for 2–4% of all primary brain tumors^[21]^. Its incidence is approximately 0.4 per 100 000 annually, increasing to 4 per 100 000 in individuals over 70 years of age^[21–23]^. Five-year survival is approximately 30–40%. Although it shows a high initial responsiveness to chemotherapy and radiotherapy, recurrence is common^[24]^. In our patient, the MATRiX chemoimmunotherapy regimen was chosen for its demonstrated high complete-response rates (over 75 %) in newly diagnosed primary CNS lymphoma^[21]^. Moreover, its four-drug combination provides synergistic antitumor activity while ensuring effective blood–brain barrier penetration^[21]^. The most common location of PCNSL is the brain parenchyma, which is reported in 92% of the patients^[25]^. Neurological symptoms such as focal deficits (50–70%), altered mental status (40–50%), and signs of increased intracranial pressure (headache, vomiting) (33%) are the most common presentation^[26,27]^. The risk factors for PCNSL include immunosuppression, EBV infection, and prior organ transplantation^[21]^. PCNSL pathogenesis involves activation of B-cell receptor signaling, immune evasion, and an immunosuppressive tumor microenvironment. Novel targeted therapies addressing these mechanisms are currently being investigated for therapeutic management^[28]^.

This report has a few limitations. Notably, no protein expression or phosphorylation assays were performed to functionally validate the impact of the identified *ZAP70* splice-site mutation because the test was not readily available at that time. However, the diagnosis was strongly supported by clinical features, profound T-cell lymphopenia, flow cytometric immunophenotyping, and confirmatory genetic testing, including Sanger sequencing. Furthermore, as a single case report, these findings may not be broadly generalizable to all individuals with *ZAP70* deficiency.

## Conclusion

*ZAP70* deficiency is a rare form of combined immunodeficiency that can predispose individuals to infections and, in some cases, malignancies, such as leukemia and lymphoma. Pathogenic variants, such as c.402 + 2 T>C may present with atypical or delayed phenotypes, including severe complications, such as EBV-driven primary CNS lymphoma. This case underscores the importance of considering *ZAP70* mutations in patients with unexplained lymphopenia and recurrent infections even in the absence of classical SCID features. Given the rarity of CNS lymphomas in *ZAP70*-deficient patients, further studies and case reports are needed to elucidate the association with CNS tumors, clarify underlying genetic and immunologic risk factors, and guide the development of targeted therapies.

## Data Availability

All data generated or analyzed during this study are included in this published article.

## Research registration unique identifying number (UIN)

Not applicable. This manuscript is a single case report and was not registered in a research registry.
